# An Unusually Large Submandibular Sialolith: A Case Report

**DOI:** 10.7759/cureus.70356

**Published:** 2024-09-27

**Authors:** Prasanna P Moon, Maithili Bankar, Sanika Kalambe, Ankit Badge, Monal M Kukde, Nandkishor J Bankar

**Affiliations:** 1 Otolaryngology - Head and Neck Surgery, Datta Meghe Medical College, Datta Meghe Institute of Higher Education and Research (Deemed to be University), Nagpur, IND; 2 Microbiology, Datta Meghe Medical College, Datta Meghe Institute of Higher Education and Research (Deemed to be University), Nagpur, IND; 3 Dentistry, Datta Meghe Medical College, Datta Meghe Institute of Higher Education and Research (Deemed to be University), Nagpur, IND; 4 Microbiology, Jawaharlal Nehru Medical College, Datta Meghe Institute of Higher Education and Research (Deemed to be University), Wardha, IND

**Keywords:** giant salivary gland calculi, megalolith, salivary gland calculi, salivary stones, sialolithiasis

## Abstract

Sialolithiasis is among the most common pathological conditions of the salivary glands. It is characterized by blockage of the salivary gland excretory duct or by the formation of calcareous concretions, resulting in salivary stasis and causing salivary gland swelling. Most sialoliths generally arise in the submandibular gland and duct. The size of sialoliths ranges from around 1 mm to 10 mm, with some exceeding 15 mm in dimension. Numerous conservative treatments, including milking and palliative therapy, are effective for accessible small stones. Surgical intervention is necessary when the sialoliths are large, inaccessible, or when conservative therapies fail. The present study reports a patient with large multiple submandibular gland sialoliths, with the largest measuring approximately 16 mm by 10 mm. This case highlights the importance of considering sialolithiasis in patients with submandibular swelling and the necessity of surgical intervention for large stones when conservative treatments fail. The patient's symptoms resolved postoperatively, demonstrating the effectiveness of the treatment approach.

## Introduction

Sialolithiasis has been reported as the most common condition affecting the salivary glands (submandibular, parotid, and sublingual), with males affected twice as often as females [1,2]. Although rare in the pediatric age group, the prevalence in children is around 3% [3]. Prolonged blockage by a sialolith can cause damage to the gland, resulting in a permanent reduction of salivary function [4]. The incidence of sialolithiasis is higher in the submandibular gland, with a rate of occurrence greater than 80%, followed by the parotid gland at 6%, and 2% in the sublingual and other minor salivary glands. Multiple calculi in the submandibular gland are rare [1]. Simultaneous calculi in multiple salivary glands are also rare [5]. Forty percent of parotid and 20% of submandibular calculi are not radiopaque, and diagnostic sialography is necessary for diagnosis in such cases. Most salivary sialoliths are unilateral and rarely cause dryness in the mouth [2]. They generally have a round to ovoid form, a smooth or rough surface, and a yellowish color. Most of the time, they are composed of calcium salts, primarily calcium phosphate, with carbonates in the form of hydroxyapatite in smaller amounts, and a smaller percentage of potassium, ammonia, and magnesium. This mixture is diffused uniformly throughout [6]. Parotid stones comprise 49% inorganic material and 51% organic material, while submandibular stones are 82% inorganic and 18% organic material [7]. Although bacterial components have not been found in the core of a sialolith, the organic material typically consists of various amino acids and carbohydrates [8].

## Case presentation

A 52-year-old male patient visited the Department of Otorhinolaryngology at Datta Meghe Medical College, Wanadongri, Nagpur, with a primary complaint of swelling on the left side of the floor of the mouth. The patient reported that the affected gland was painful and swollen, especially during mealtimes and in response to other salivary stimuli. The patient also complained of a foreign body sensation beneath the tongue for the past three months, which was insidious in onset, gradually progressive, and continuous in nature. There was no history of fever. The patient reported no family history of a similar disease and no history of a similar condition in the past.

Clinical findings

Bimanual palpation revealed a large, firm swelling measuring 2 cm x 3 cm in diameter, located on the left side of the floor of the mouth, in the region of the opening of the submandibular duct. The swelling had irregular borders, a smooth surface, and signs of inflammation were present. On palpation, it was tender, but the local temperature was not raised. Cervical lymph nodes were not palpable. The consistency of the swelling was hard and not fixed to the mucosa. Other intraoral findings included carious teeth with calculus and stains. Investigations included a complete hemogram, random blood sugar levels, and an X-ray of the submandibular region. Table 1 shows routine hematologic evaluation was within normal limits.

**Table 1:** Routine hematologic evaluation Hb: hemoglobin; RBC: red blood cell; MCV: mean corpuscular volume; MCH: mean corpuscular hemoglobin; MCHC: mean corpuscular hemoglobin concentration; WBC: white blood cell; g/dL: grams per deciliter.

Test	Result	Reference Range
Hb	14.60 g/dL	13.8-17.2 g/dL
Hematocrit	43%	40-52%
RBC	4.9 million cells/µL	4.5-5.9 million cells/µL
MCV	86 fL	80-100 fL
MCH	29.03 pg	27-31 pg
MCHC	34.06 g/dL	32-36 g/dL
WBC	6400 cells/µL	4,000-11,000 cells/µL
Platelet count	320,000 cells/µL	150,000-450,000 cells/µL
Neutrophils	54%	40-60%
Lymphocytes	35%	20-40%
Monocytes	6%	2-8%
Eosinophils	3%	1-4%
Basophils	0.6%	0.5-1%
Reticulocyte count	1.4%	0.5-2.5%

**Figure 1:**
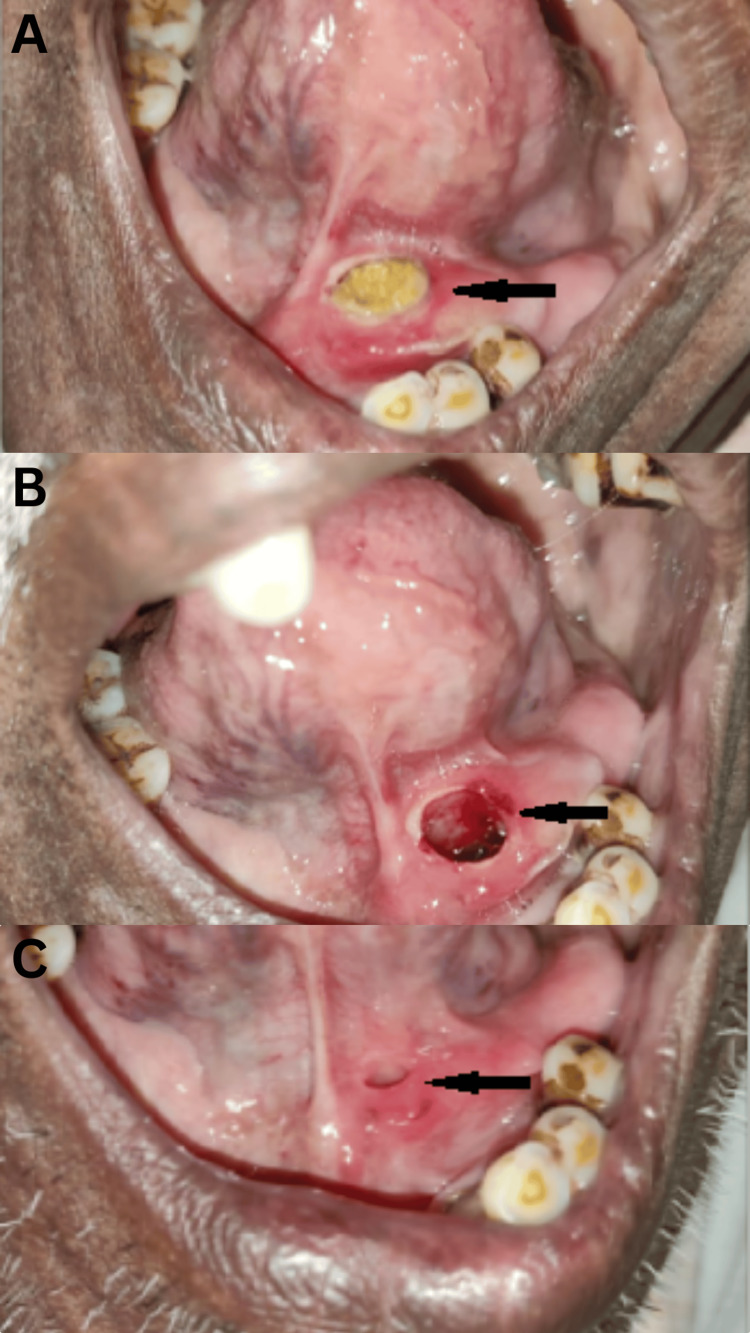
Surgical images of submandibular duct (A) The black arrow shows the pre-operative left submandibular duct with calculus inside the duct. (B) The black arrow shows the operative left submandibular duct opening after the removal of the calculus. (C) The black arrow shows the post-operative left submandibular duct.

Figure 1 shows pre-operative (A), operative (B), and post-operative (C) views of the left submandibular duct with calculus removal.

**Figure 2:**
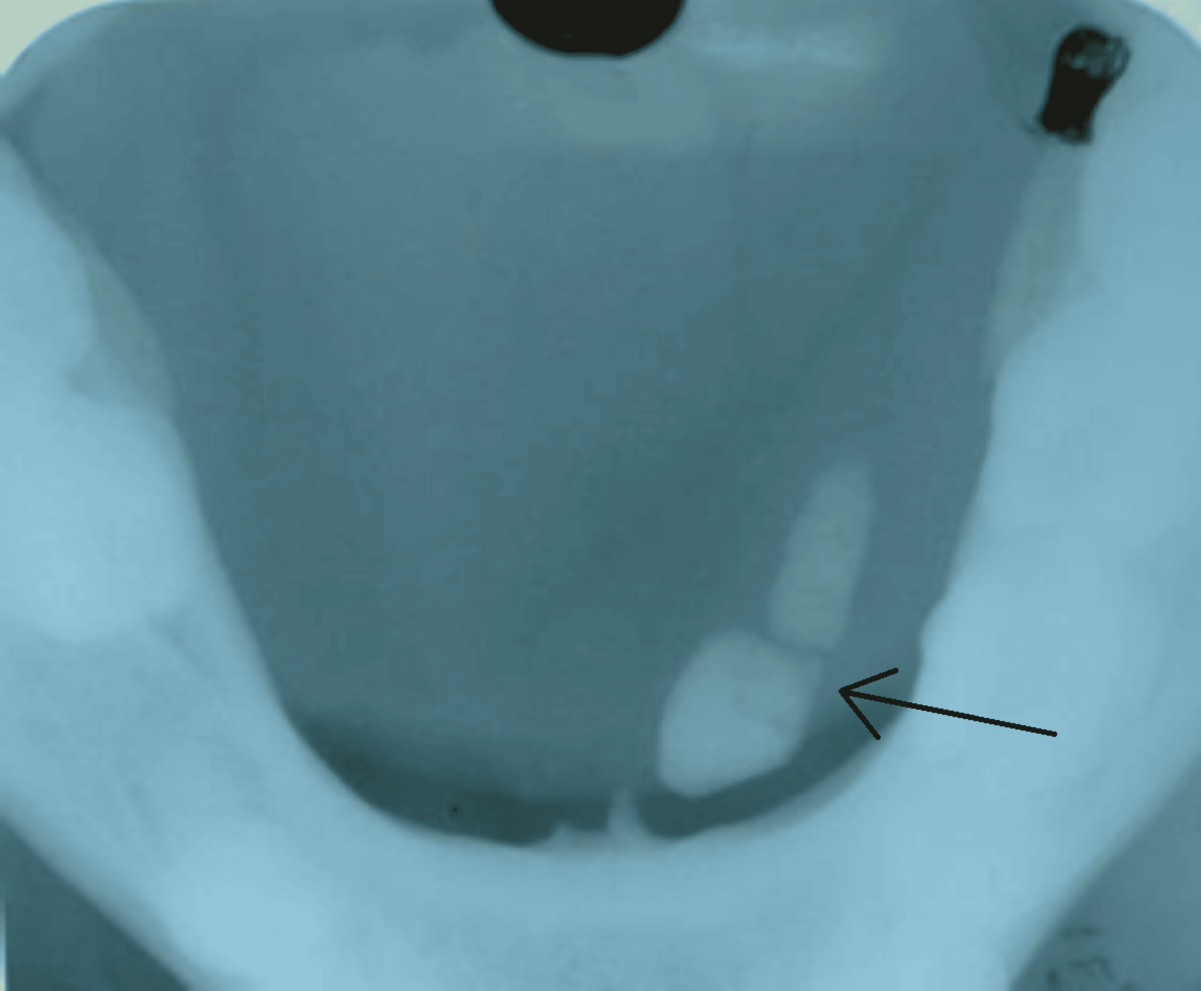
Mandibular occlusal radiograph shows round to oval radiopaque submandibular salivary stone (black arrow) located left side on floor of mouth

Figure 2 shows an occlusal radiograph showing a round to oval radiopaque sialolith, measuring approximately 1.5 cm x 2 cm, confirming the diagnosis of submandibular sialolithiasis.

Diagnosis

Diagnosis of salivary calculi was confirmed by medical history, physical examination, and X-ray. A purulent draining discharge was observed from the salivary duct.

Treatment

**Figure 3:**
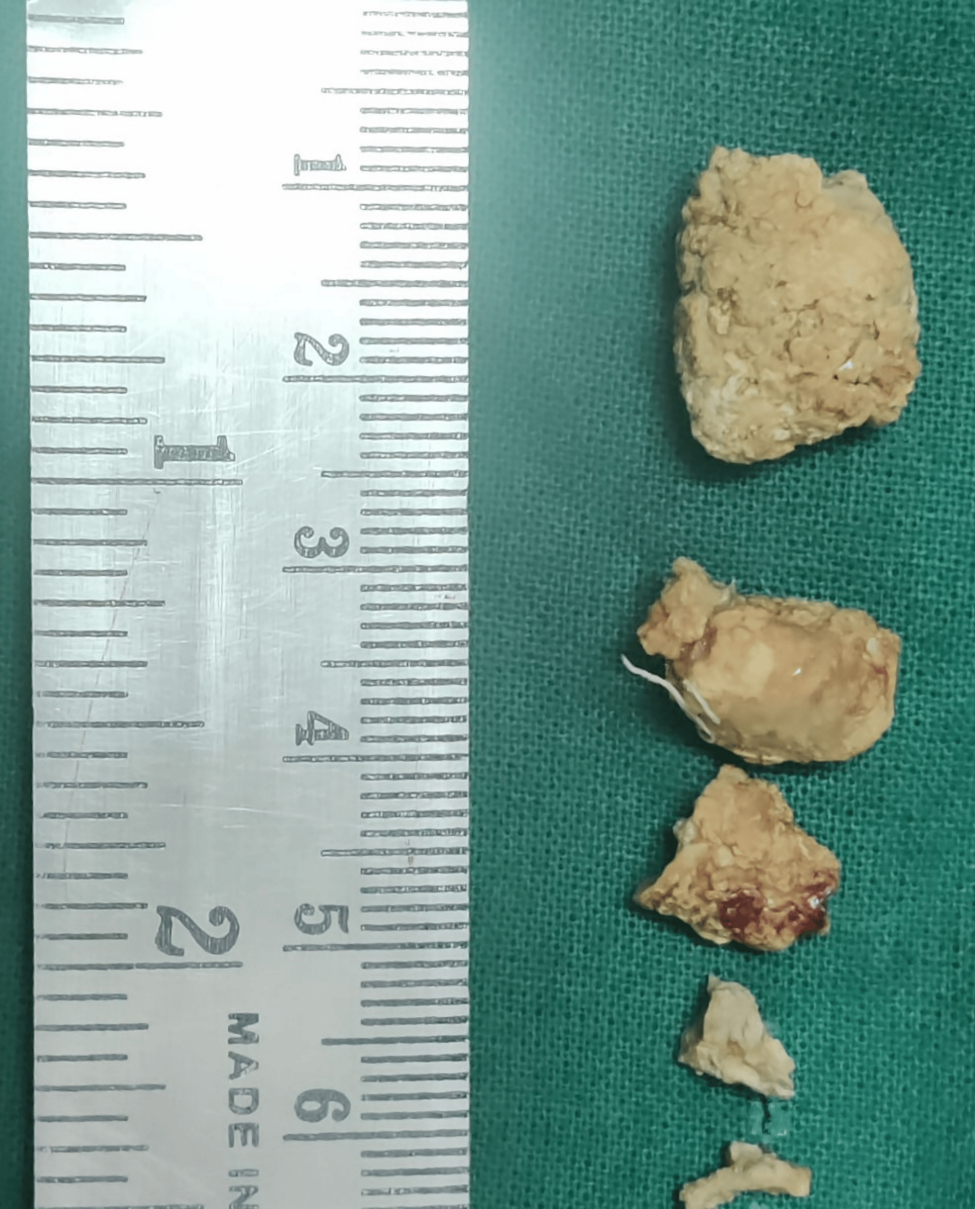
Multiple submandibular salivary stone removed from the left submandibular gland

A left submandibular duct calculus was identified as the cause, and the diagnosis was made based on clinical and radiological examination. The stones were retrieved under 10% lignocaine local anesthesia. Multiple stones were removed, the largest measuring 1.5 cm x 2 cm. Dilatation of the salivary duct of the left submandibular gland was observed after the removal of the stones. The non-steroidal anti-inflammatory drug ibuprofen, 400 mg thrice daily, was administered for five days to reduce pain and swelling. The antibiotic amoxicillin-clavulanic acid, 625 mg twice daily, was administered for five days. Betadine gargles were recommended to maintain oral hygiene and prevent recurrence. Figure 3 shows the multiple stones that were removed from the salivary duct of the left submandibular gland.

Follow-up

One week after the procedure, clear saliva was observed from the left submandibular gland duct. After one month of follow-up, the patient did not have pain in the submandibular region with clear salivary flow.

## Discussion

Sialoliths are formed by the deposition of tricalcium phosphate salts combined with mucus, bacterial nidus, or desquamated cells. Calculus development may be accelerated by increased salivary alkalinity and calcium concentration, as well as by inflammation, infection, or damage to the salivary gland or its duct. Due to the higher calcium and phosphate content of saliva and the increased alkaline pH, the submandibular glands are more prone to developing calculi [9,10]. Chemical, neurogenic, inflammatory, mechanical, and infectious causes are all etiological factors in the development of salivary gland calculi [11]. Calculi depend on the relative stagnation of calcium-rich saliva [2]. They are formed when calcium salts settle around an initial organic nidus that contains bacteria, desquamated epithelial cells, and altered salivary mucins [9]. The mucoid component of saliva changes due to intermittent stasis, producing a gel necessary for stone formation. This gel creates the framework for the accumulation of salts and organic materials that lead to stone formation [12]. According to conventional theories, stone formation occurs in two phases: a central core and a layered periphery of organic and non-organic substances [13]. The central core is made up of salt precipitants, which are bound by chemical and organic substances [14].

Submandibular sialoliths are expected to form around a mucous nidus, while parotid stones are believed to frequently form around a nidus of inflammatory cells or a foreign object [15]. Another theory contends that an unidentified metabolic process can raise salivary bicarbonate levels, affecting the solubility of calcium phosphate and causing calcium and phosphate ions to precipitate [8,16]. Food particles or germs in the mouth can move into the salivary ducts, serving as a nidus for further calcification [1]. Calculus formation is predisposed by increased salivary alkalinity, stasis, infection or inflammation, and physical trauma to the salivary gland or its duct [17]. Because submandibular saliva has a higher concentration of phosphate and calcium, is more mucous than the saliva of the parotid gland, and is more alkaline, submandibular sialolithiasis is more frequent [18].

The submandibular duct is also longer and has an antigravity flow [17]. Systemic calcium metabolism abnormalities are not associated with stone formation [19]. Studies on parathyroid hormone and electrolytes in people with sialolithiasis have not revealed any abnormalities [20]. Gout is the only recognized systemic condition that increases the risk of developing salivary stones, with uric acid being the primary component of gout stones [21]. Pachisia S et al. [1] reported a case of sialolith diagnosed solely by X-ray. Thus, uncomplicated sialolith can be diagnosed by x-ray alone, particularly in resource-constrained settings or when the patient has limited financial means.

## Conclusions

Sialolithiasis, particularly in the submandibular gland, can cause significant discomfort and requires timely intervention. Surgical removal is effective for large, inaccessible stones. A thorough history taking and complete examination are necessary to support the clinical diagnosis, and it can be diagnosed with an X-ray alone in resource-constrained settings. Infection should be suspected in the presence of gland enlargement and sialolithiasis. Early diagnosis and treatment are crucial to prevent glandular damage and ensure complete recovery.
